# Robust Tracking of a Cost-Effective Micro-Stereolithography System Based on a Compliant Nanomanipulator †

**DOI:** 10.3390/mi10110785

**Published:** 2019-11-16

**Authors:** Yue Cao, Zhen Zhang

**Affiliations:** 1 State Key Laboratory of Tribology & Institute of Manufacturing Engineering, Department of Mechanical Engineering, Tsinghua University, Beijing 100084, China; y-cao18@mails.tsinghua.edu.cn; 2 Beijing Key Laboratory of Precision/Ultra-Precision Manufacturing Equipment and Control, Tsinghua University, Beijing 100084, China

**Keywords:** micro-stereolithography (MSL), compliant nanomanipulator, tracking

## Abstract

Micro-stereolithography (MSL) has emerged as a promising and challenging technique in micro-/nano-scale additive manufacturing. Besides the requirement of the light source, the motion system requires ultra-high-precision tracking capability to reach the right location for every solidification event. To achieve single-digit micron feature size of the fabrication, we propose a robust control strategy to support a self-developed cost-effective MSL prototype based on a compliant nanomanipulator and a blue light-emitting diode (LED) module. In particular, the nonlinearity and parameter-variation of the compliant manipulator are dealt with by a robust radial basis function (RBF)-based neural network, and the repetitive control (RC) is innovatively integrated with RBF to improve the tracking performance of a closed pattern. Various simulations and real-time experiments are conducted to validate the proposed control strategy. The fabrication of a closed pattern will not begin by turning on the laser source until the tracking error reaches submicrons, and the fabrication results demonstrate that the cost-effective MSL system is capable of fabricating 2.5 µm feature size in a 0.5 mm working range.

## 1. Introduction

The progress of additive manufacturing (AM) technologies has significantly improved the capability of manufacturing 3D microstructures with complicated geometries. Among AM technologies, stereolithography (SL) and micro-stereolithography (MSL) [1,2,3] (i.e., SL in micro-scale) are widely utilized in many domains, owing to the high resolution, precision, and adaptability of the increasing number of materials. Specifically, these developments enable the precise fabrication of biomaterial scaffold [4], bioprinting [5], and microfluidic devices [6]. With the advance of MSL, more stringent fabrication demands are posed. For example, a micro-tweezer in [7] requires 2 µm accuracy and 400 µm working range, and a microscopic four-point probe of a microsensor in [8] requires 200 nm accuracy and a 10 mm working range.

To improve the fabrication quality of MSL processes, the investigation can be mainly classified by two threads. One thread is to improve the laser sources and photopolymer. In this respect, the two-photon polymerization process was developed in [9,10], of which the resolution was around 100 nm within 20 µm working range. Nevertheless, the technique requires costly two-photon laser sources and complex system setups. Besides the two-photon polymerization method, the nonlinearity of the photopolymer was utilized to improve the accuracy of MSL fabrication [11,12], where the wavelength of the laser source was very restrictive, resulting in complex optical transferring systems and costly setups.

Another thread is improving the motion systems of MSL processes. Since continuous motion is required during MSL fabrication, the dynamics of the motion system is very important to achieve high-precision motion quality. Recent MSL apparatuses based on cost-effective light sources utilized commercial motion stages, such as [13,14], showing ∼20 µm linewidth. As a result, it was difficult to interfere in the drivers of commercial motion stages, and hence the system dynamics cannot be dealt with by self-designed control methods for specific microstructures.

With the above discussion, we, in this paper, would like to propose a robust tracking control strategy for a self-developed compliant nanomanipulator-based, cost-effective MSL system, so that a pattern of ∼2 micron feature size can be formed by solidifying a liquid photopolymer in a layer. For a compliant mechanism, an increased stroke usually leads to a more significant stiffness nonlinearity, and the parameter-varying nonlinear terms will be not negligible as well. Those problems can be dealt with by adaptive control [15,16] or neural network control [17], which is considered an effective way to approximate nonlinear functions. Compared with the back propagation (BP) network [18], the radial basis function (RBF) network is more widely used because of its high adaptability, simple network structure, and low computation load. It is noted that the RBF network is capable of approaching any nonlinear function at a reasonable precision within a compact set [19].

In the proposed control strategy, the baseline tracking controller is a RBF network-based control unit, and a plug-in repetitive controller is integrated to improve the tracking performance for a closed contour. The main contributions of this paper are as follows:

1. The proposed RBF-based robust tracking control strategy is capable of dealing with unknown and complex nonlinear dynamics, and repetitive control (RC) is innovatively integrated with RBF to improve the tracking performance of closed patterns for a compliant manipulator.
2. With the proposed control applied on a self-developed cost-effective MSL system, the fabrication of a closed pattern will not be started (by switching on the laser source) until the tracking error reaches to submicrons. The fabrication results demonstrate that the cost-effective MSL system is capable of fabricating 2.5 µm feature size in a 0.5 mm working range.

The rest of the paper is organized as follows. The system description of a cost-effective MSL apparatus is presented in Section 2. A robust tracking control design of the MSL system is proposed in Section 3. To demonstrate the proposed control strategy, various simulations and experiments are conducted in Section 4, followed by some conclusions in Section 5.

## 2. System Description of a Cost-Effective MSL Apparatus

**Figure 1:**
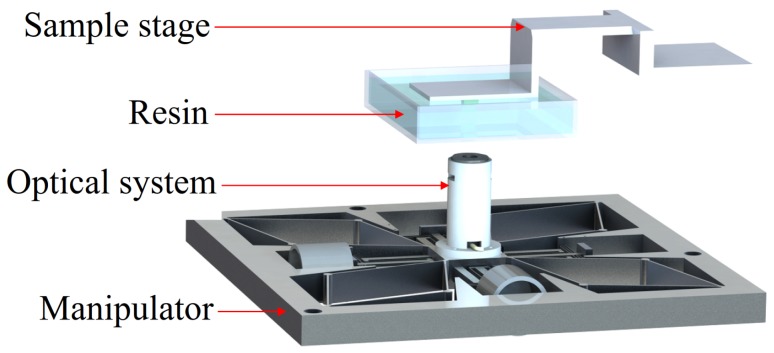
Schematics of the micro-stereolithography (MSL) system based on the fixed-surface method.

The lab-made MSL system utilized the fixed surface method (top-down structure) by which the sample is fixed. In the optical system, a blue laser diode module (wavelength: 405 nm) was utilized for its compactness and low cost. For the above system structure, the sample was immersed in an SU-8 polymer resin and mounted on a Z-axis motion stage, and the laser module was mounted on an XY motion stage. Since the MSL is operated in a layer-by-layer fashion, it is crucial to achieve a 2-D profile. Provided the 2-D trajectory with a desired accuracy, a 3-D lithography object can be obtained with the Z-axis motion. A schematic of the MSL system is shown in Figure 1. As a result, this compact structure can reduce vibration and avoid planar motion between the sample and polymer resin during the fabrication.

**Figure 2:**
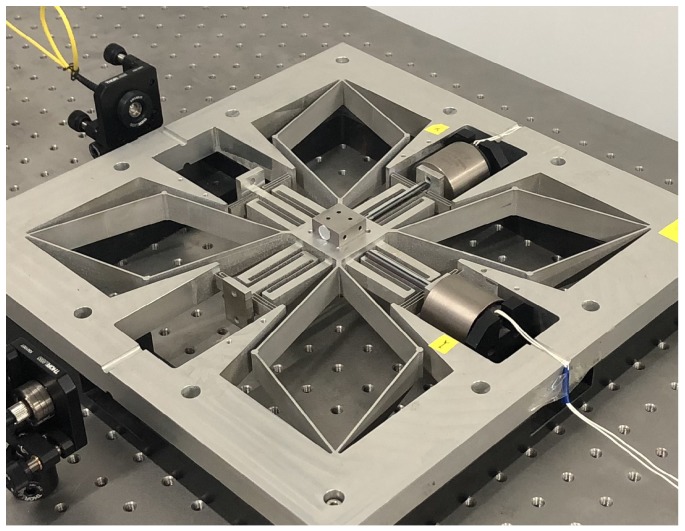
An XY compliant nanomanipulator.

In particular, we realize the above XY motion stage by a self-developed compliant nanomanipulator, due to its nano-precision motion quality and millimeter motion range. The self-developed compliant nanomanipulator shown in Figure 2 serves as the XY motion of the MSL system, which is of the dimension 330 mm × 330 mm × 30 mm. The X and Y axes of the manipulator are actuated by voice coil motors. The working range of this manipulator is up to ±1.5 mm × ±1.5 mm. The detailed mechanical design is referred to in [20]. Thanks to the flexure bearings, friction and backlash can be avoided during the operation, and no maintenance is required. In addition, the Z-axis motion is achieved by a single-axis motion stage actuated by a rotary motor, because the precision requirement of a layer-by-layer motion is not strict.

**Figure 3:**
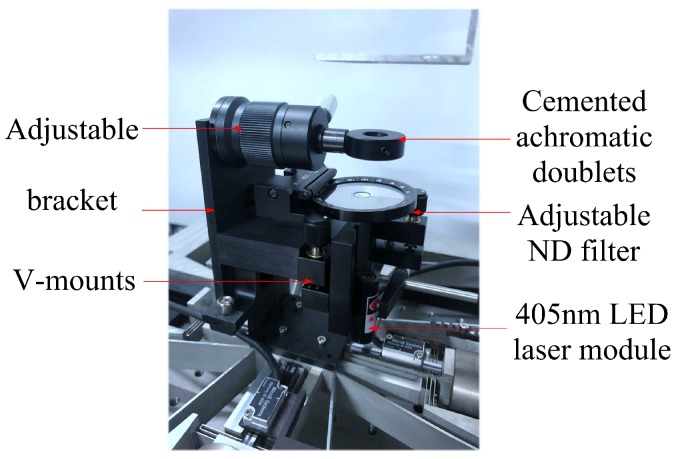
A cost-effective and compact optical system.

The compact laser source module shown in Figure 3 is composed of a 405 nm LED, an APC circuit, an ACC circuit, and a focusing lens. The laser beam was focused by cemented achromatic doublets (GCL-010613, Daheng Optics Co., Beijing, China) after passing through an adjustable neutral-density (ND) filter (GCC-303004, Daheng Optics Co., Beijing, China). In addition, kinematic V-mounts (GCM-182101M, Daheng Optics Co., Beijing, China) was adopted to fix the laser module, and an adjustable height post (GCM-0101M, Daheng Optics Co., Beijing, China) was adopted for the doublets to achieve precise alignment of the optics. The overall size of the optical system is about 50 mm × 70 mm × 120 mm. The focused spot size by the optical system is around 3 microns.

**Figure 4:**
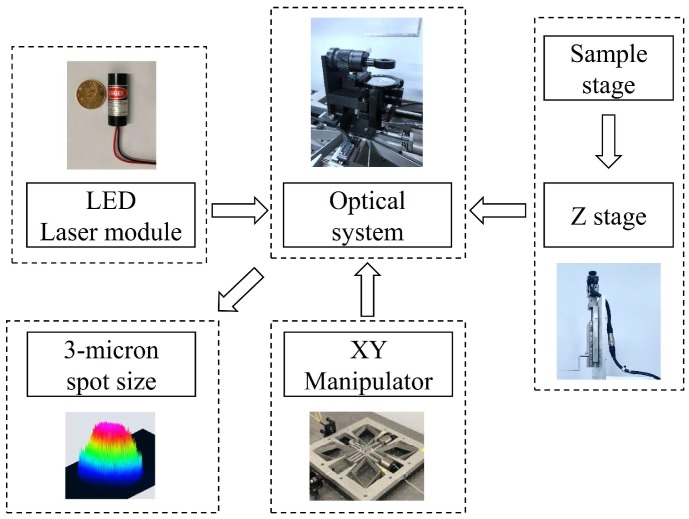
A schematic of an MSL system.

By combining the above XY nanomanipulator and Z motion stage as well as the optical system, we built an MSL prototype, shown in Figure 4.

## 3. Robust Control Design

To precisely control the motion of such a manipulator, we need to obtain its dynamic model.

### 3.1. Dynamic Model of the Compliant Nanomanipulator

**Figure 5:**
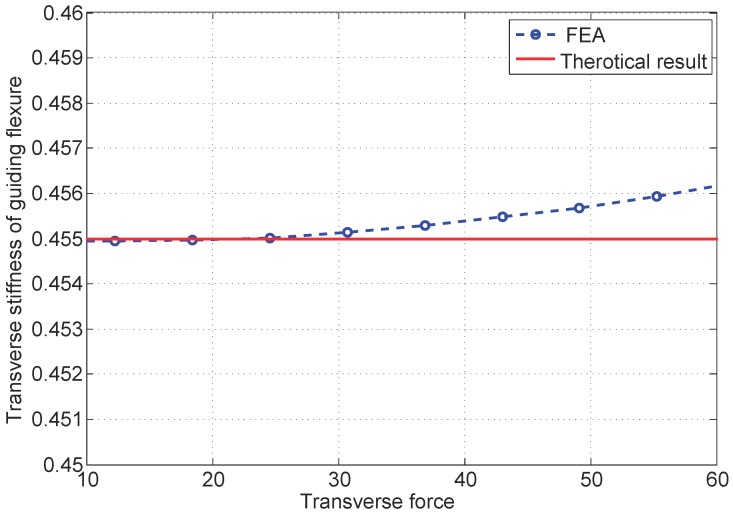
The normalized stiffness of the guiding flexure.

Note that in a relative small range, the damping and stiffness of the manipulator can be regarded as constants, so the linear model can be utilized to represent its major dynamics. Nevertheless, it is worth noting that the working range of the MSL may not be small in practice. In a relatively large range (over 0.5 mm), the nonlinearity of the stiffness cannot be neglected, and the explicit form is very difficult to express, but its finite element analysis (FEA) can be shown in Figure 5.

From Figure 5, it is seen that the stiffness of the compliant manipulating system is a nonlinear function of acceleration, depending on the force. Meanwhile, the force coefficient *b* of voice coil actuators depends on the displacement. In addition, when the system is in planar motion, the cross-axis coupling also leads to nonlinearity. With this, the dynamics of the manipulator in one axis can be expressed as follows:

(1) $$\begin{array}{ccc} \dot{x} & = & \left[\begin{array}{cc} 0 & 1\\ 0 & 0 \end{array}\right]x+\left[\begin{array}{c} 0\\ 1 \end{array}\right]\left(\alpha \left(x,\;\bar{x}\right)+\beta \left(x,\;\bar{x}\right)u+d\left(t\right)\right)\\ y & = & \left[\begin{array}{cc} 1 & 0 \end{array}\right]x\;, \end{array}$$

where $x={\left(x_{1}\;x_{2}\right)}^{T}$ is the state of one axis, and $\bar{x}={\left({\bar{x}}_{1}\;{\bar{x}}_{2}\right)}^{T}$ is the state of another axis, and u∈R and y∈R are the control input and output, respectively, and nonlinear functions α(·,·) and β(·,·) collect overall nonlinear terms and cross-axis coupling terms, and $|\beta \left(x,\bar{x}\right)|\leq \beta_{0}$, and d(t) is the disturbance satisfying $\left|d\left(t\right)\right|\leq d_{0}$.

The above nonlinear dynamics pose a challenge for a robust control design, which will be discussed in the following section.

### 3.2. Robust Adaptive Control Based on Radial Basis Function (RBF) Neural Network

To tackle the challenge of parameter-varying nonlinear functions in the system (1), we considered an RBF neural network, due to its good capability of approximating nonlinearity. Moreover, during the operation of the MSL system, the load varies under different working conditions, and a mass eccentricity effect exists. Compared with other controllers, the RBF-based network requires less tuning procedures, which facilitates the operation of the MSL system.

The RBF network contains three layers: the input layer, hidden layer, and output layer. The activation function of neurons in the hidden layer is composed of radial basis function. The arithmetic unit consisting of the hidden layer is called the hidden layer node. Each hidden layer node contains a central vector *c*, and *c* shares the same dimension with the input vector *p*. The Euclidean distance between them is defined as $∥p\left(t\right)-c_{j}\left(t\right)∥$. The output of the hidden layer is a nonlinear activation function $h_{j}\left(t\right)$, which can be written as:

(2) $$h_{j}\left(t\right)=\exp\left(-\frac{{∥p\left(t\right)-c_{j}\left(t\right)∥}^{2}}{2b_{j}^{2}}\right)\;,\;j=1,\cdots ,l,$$

where $b_{j}$ is a positive scalar representing the width of the Gaussian basis function, and *l* is the number of nodes of the hidden layer. The output of the network is enabled by the following weighting functions:

(3) $$q_{i}\left(t\right)=\sum_{j=1}^{m}w_{ji}h_{j}\left(t\right)\;,\;i=1,\cdots ,n,$$

where *w* is the weight of the output layer, and *n* is the number of output nodes, and *q* is the output of the neural network.

Recall the nonlinear parameter-varying system (1). Set *r* as the reference, then the tracking error $e=x_{1}-r$ and an error function $s=\lambda e+\dot{e}$ with λ>0. Let $z={\left(x\;\bar{x}\;s\;v\right)}^{T}$ be the input vector of the RBF neural network to realize a robust adaptive control. Let $\hat{W}$ be the estimate of ideal network weights $W^{*}$, and then the control input can be designed as the output of the RBF network—that is:

(4) $$u={\hat{W}}^{T}h\left(z\right),$$

where $h\left(z\right)={\left(h_{1}\left(z\right)\;h_{2}\left(z\right)\;\cdots \;h_{l}\left(z\right)\right)}^{T}$. The adaptive law is chosen as:

(5) $${\dot{\hat{W}}}^{T}h\left(z\right)=-\Gamma \left(h\left(z\right)s+\sigma \hat{W}\right),$$

where $\Gamma =\Gamma^{T}>0$ is the adaptive gain matrix, and σ>0 is a constant. The certainty-equivalence controller

(6) $$u^{*}=-\frac{1}{\beta }\left(\alpha +v\right)-\left(\frac{1}{\varepsilon \beta }+\frac{1}{\varepsilon \beta^{2}}-\frac{\dot{\beta }}{2\beta^{2}}\right)s\;,$$

with parameter ε. Substitute control law (5) into $\dot{s}$, the error function dynamics reads as

(7) $$\begin{array}{c} \dot{s}=\alpha +v+\beta \left({\hat{W}}^{T}h\left(z\right)-{\hat{W}}^{*T}h\left(z\right)-\mu_{l}\right)+\beta u^{*}+d\left(t\right)\;, \end{array}$$

where $v=\lambda \dot{e}-\ddot{r}$, and $\mu_{l}=u^{*}-{\hat{W}}^{*T}h\left(z\right)$ is the network estimation error of $u^{*}$. Substitute Equation (6) into Equation (7), yielding

(8) $$\dot{s}=\beta \left({\tilde{W}}^{T}h\left(z\right)-\mu_{l}\right)-\left(\frac{1}{\varepsilon }+\frac{1}{\varepsilon \beta }-\frac{\dot{\beta }}{2\beta }\right)s+d\left(t\right),$$

where $\tilde{W}=\hat{W}-W^{*}$.

The boundness of $\hat{W}$ and *s* is provided in the following result.

**Theorem** **1.** *Consider system (1). If the network’s estimation error $|\mu_{l}|\leq \mu_{0}$, then the controller (4) renders the weights estimation $\hat{W}$, and the error function s is bounded.*

**Proof.**  See the Appendix A. □

**Remark** **1.** *The bound of s is independent of reference r, and by appropriately tuning the parameters of the neural network, the tracking error can be made arbitrarily small.*

### 3.3. An Add-On Structure of Plug-In RC

With the RBF-based robust adaptive control, the robust tracking is achieved in the presence of an unknown parametric nonlinear system. Note that the controller is designed for the entire frequency band. We would like to further optimize the tracking performance for periodic trajectories because a closed contour can be repeated to form a periodic trajectory during an MSL fabrication.

**Figure 6:**
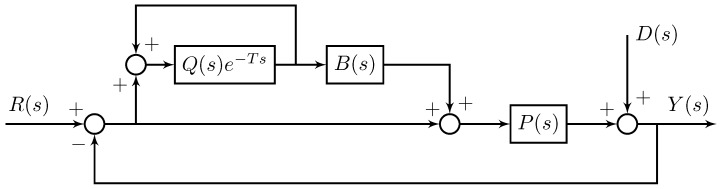
The structure of plug-in repetitive control (RC).

For the sake of periodic tracking, we applied the plug-in repetitive control (plug-in RC) [21] to the RBF-based robust adaptive control system. As shown in Figure 6, the compensator B(s) needs to include the inverse of the complementary sensitivity of P(s). Since the tracking is achieved by the proposed RBF robust adaptive control, the complementary sensitivity function approximately equals to 1. With this, let B(s)=1. Also, the low-pass filter Q(s) is designed to enhance high-frequency attenuation and to offset high-frequency resonance, as well as to improve the transient response of the closed-loop system. In this work, a first-order low-pass filter $Q\left(s\right)={\left(\tau s+1\right)}^{-1}$ was designed.

**Remark** **2.** *The plug-in RC control only adjusts the input $\left(x,\;\bar{x}\right)$ to the RBF neural network, so the stability analysis remains the same as that in Theorem 1.*

**Figure 7:**
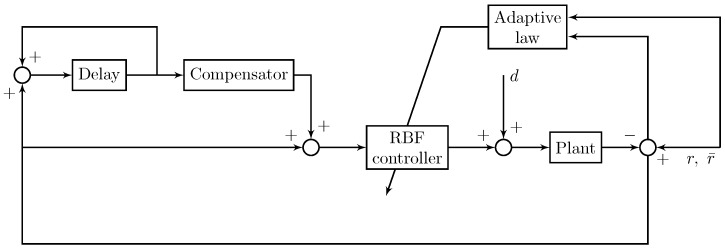
The block diagram of the proposed robust tracking control strategy.

By integrating the plug-in RC with the RBF-based robust adaptive control, the proposed integrated controller can be obtained as shown in Figure 7.

## 4. Simulation and Experimental Results

### 4.1. Simulation Results

In the simulation, the following identified transfer functions (9) of each axis of the manipulator without varying parameters is used as the nominal plant model,

(9) $$\begin{array}{cc} P_{x}\left(s\right) & =\frac{548248.13}{s^{2}+14.88s+101793.23}\\ P_{y}\left(s\right) & =\frac{319852.42}{s^{2}+18.52s+99664.23}\;. \end{array}$$

**Figure 8:**
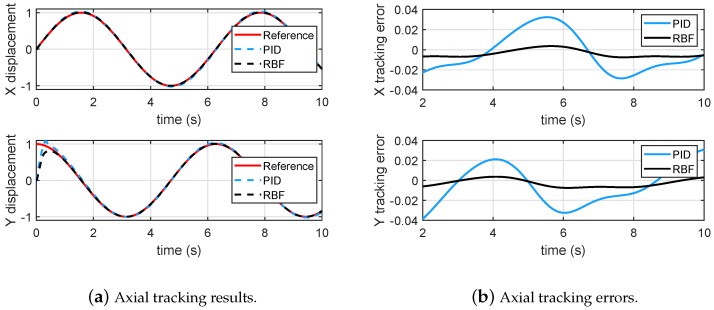
Tracking of a circular trajectory.

The nonlinear terms $\alpha =x_{1}^{2}+x_{1}{\bar{x}}_{2}$, and $\beta =\beta_{l}+x_{2}^{2}-x_{1}^{2}{\bar{x}}_{2}$ are introduced to represent unknown parametric nonlinearity, where $\beta_{l}$ is the linear part which can be obtained from (9). The initial state is set to zero. Specifically, a circular trajectory tracking is conducted, and Figure 8a,b shows that the proposed RBF controller significantly outperforms the PID controller in the presence of an unknown parametric nonlinearity.

**Figure 9:**
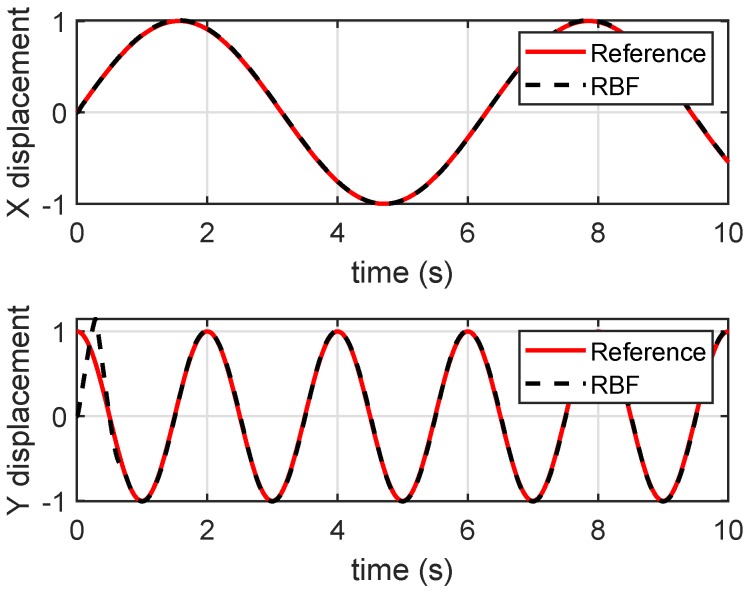
Tracking results when references were without cross-correlation.

To validate the approximation performance of the RBF on the nonlinear function, we removed the cross-correlation between X and Y axes by taking sinusoidal signals of irreducible frequencies as the references for each axis. Figure 9 shows the RBF-based control remains a good tracking performance in the absence of the cross-correlation of X and Y axes.

**Figure 10:**
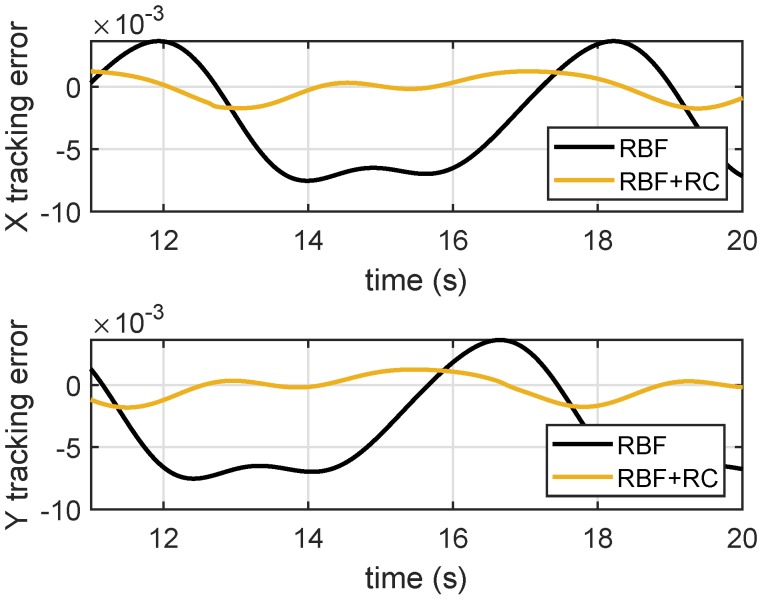
Axial tracking errors of integrated RC and RBF control.

Furthermore, to validate the performance of the plug-in RC controller for periodic tracking, we added the plug-in RC to the RBF-based robust adaptive controller. Figure 10 shows that the RC controller significantly reduces the tracking errors.

### 4.2. Experimental Results

**Figure 11:**
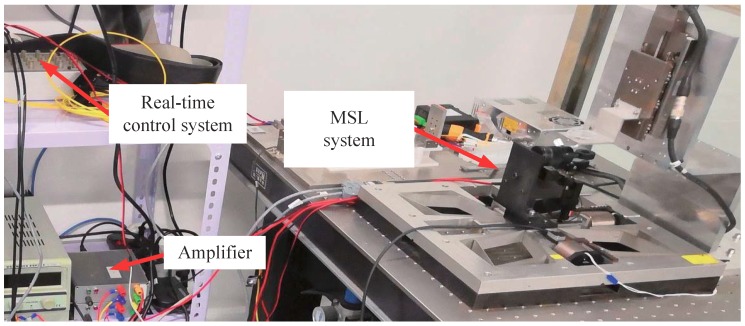
Experimental setup.

**Figure 12:**
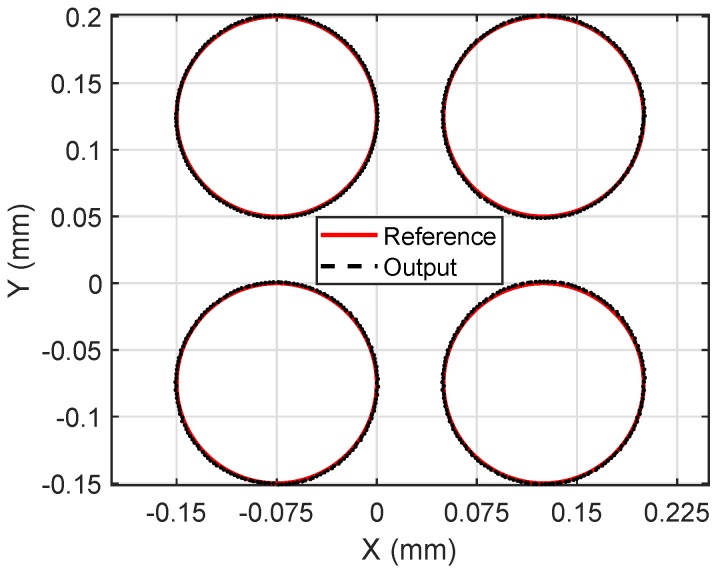
Tracking results of the experiment.

**Figure 13:**
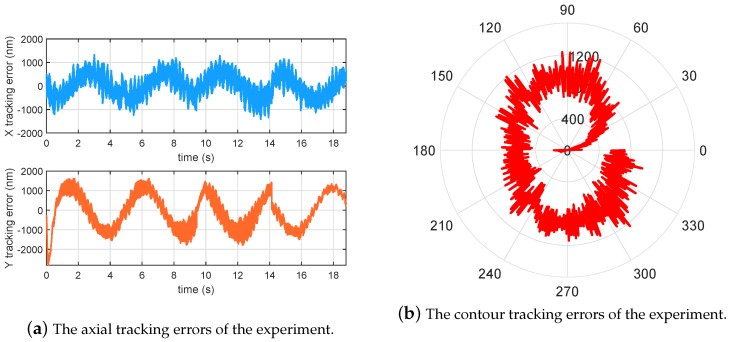
The error analysis of the experimental results.

In this subsection, experiments of the proposed robust tracking control strategy have been conducted for the self-developed MSL system shown in Figure 11. A circular contour with diameter of 0.15 mm was fabricated by the MSL system. To testify the repeatability of fabrication, four circle patterns were fabricated during one operation. The working range of the manipulator was about 0.5 mm. The fabrication process cannot be started by switching on the laser source until the tracking error reaches submicrons in 3–4 periods. The experimental result is shown in Figure 12, and the tracking errors of the manipulator are provided in Figure 13a, showing that the axial tracking errors of the X and Y axes are 485.8 nm and 885.8 nm, respectively. Meanwhile, the contour errors of the four circles are averaged and plotted in Figure 13b.

**Figure 14:**
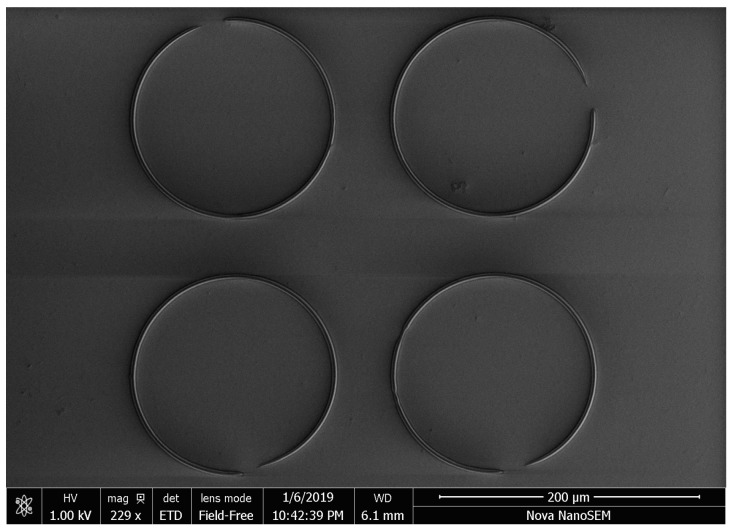
The SEM image of the MSL fabrication result.

**Figure 15:**
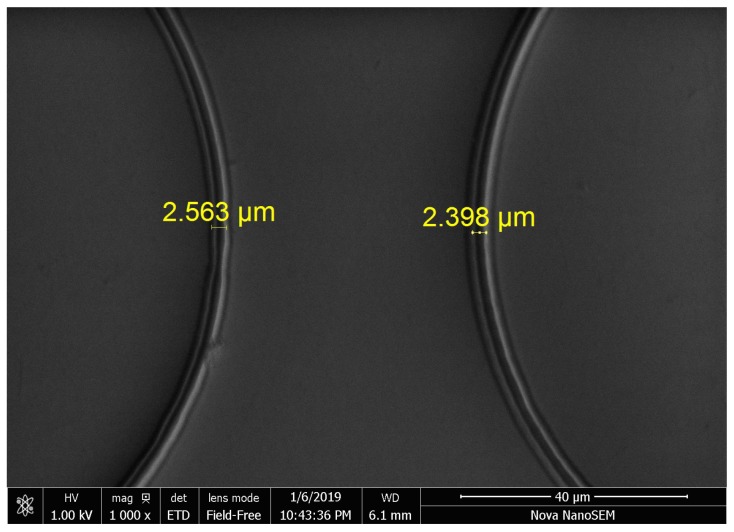
The SEM image of the fabricated feature size.

It is noted from Figure 13b that the contour error starts from zero, as there is a pause before fabricating every circle. As shown in Figure 13b, the contour errors 769.5 nm (RMS) are relatively uniform when the phase varies, which is a satisfactory result for practical MSL fabrication. The SEM image of the fabrication result is shown in Figure 14, where the linewidth of the fabricated pattern is of ∼2.5 µm in Figure 15. Note from Figure 14 that some portions of the fabricated circles are not closed. This is caused by the warm-up time of the laser source module. Although the laser source is accurately switched on and synchronized with the motion system, it needs a few microseconds to warm up until its power reaches the threshold for the lithography. The laser time delay can be compensated by software in the future.

## 5. Conclusions

In this paper, we have proposed a robust control strategy to support a cost-effective MSL prototype based on a compliant nanomanipulator and a blue LED module. Thanks to a robust RBF neural network, the nonlinearity and parameter variation of the manipulator has been successfully handled. The tracking performance of a closed pattern is enhanced by the integration of the repetitive control and the RBF-based control. Real-time experiments of the proposed robust tracking control strategy on the MSL prototype demonstrate the fabrication capability of a 2.5 µm feature size in a 0.5 mm working range. The fabrication of 3D geometries is currently under investigation.

## Appendix A

**Proof** **of** **Theorem** **1.** Choose a Lyapunov function candidate

(A1) $$V=\frac{1}{2}\left(\frac{s^{2}}{\beta }+{\tilde{W}}^{T}\Gamma^{-1}{\tilde{W}}^{T}\right).$$

Take the derivative on Equation (A1) and combine the result with Equation (8), yielding

(A2) $$\dot{V}=-\left(\frac{1}{\varepsilon \beta }+\frac{1}{\varepsilon \beta^{2}}\right)s^{2}+\frac{d(t)}{\beta }s-\mu_{l}s-\sigma {\tilde{W}}^{T}\hat{W}.$$

By noting that

$$\begin{array}{cc} 2{\tilde{W}}^{T}\hat{W} & =∥\tilde{W}{∥}^{2}+∥\hat{W}{∥}^{2}-∥W^{*}{∥}^{2}\geq ∥\tilde{W}{∥}^{2}-{∥W^{*}∥}^{2}\\ \frac{d(t)}{\beta }s & \leq \frac{s^{2}}{\varepsilon \beta^{2}}+\frac{\varepsilon }{4}d^{2}\left(t\right)\\ |\mu_{l}s| & \leq \frac{s^{2}}{2\varepsilon \beta^{2}}+\frac{\varepsilon }{2}\mu_{l}^{2}\beta \leq \frac{s^{2}}{2\varepsilon \beta^{2}}+\frac{\varepsilon }{2}\mu_{l}^{2}\beta_{0}\;, \end{array}$$

we have

(A3) $$\dot{V}\leq -\frac{s^{2}}{2\varepsilon \beta^{2}}-\frac{\sigma }{2}∥\tilde{W}{∥}^{2}+\frac{\varepsilon }{2}\mu_{0}^{2}\beta_{0}+\frac{\varepsilon }{4}d_{0}^{2}+\frac{\sigma }{2}{∥W^{*}∥}^{2}.$$

Since ${\tilde{W}}^{T}\Gamma^{-1}\tilde{W}\leq \bar{\gamma }{∥\tilde{W}∥}^{2}$, we have

(A4) $$\dot{V}\leq -\frac{1}{\alpha_{0}}V+\frac{\varepsilon }{2}\mu_{0}^{2}\beta_{0}+\frac{\varepsilon }{4}d_{0}^{2}+\frac{\sigma }{2}{∥W^{*}∥}^{2},$$

where $\bar{\gamma }$ is the largest eigenvalue of $\Gamma^{-1}$, and $\alpha_{0}=\max\left\{\varepsilon ,\bar{\gamma }/\sigma \right\}$. Solve the inequality by applying the result in [22], yielding

(A5) $$\begin{array}{c} V\left(t\right)\leq e\left(-\frac{t}{\alpha_{0}}\right)V\left(t_{0}\right)+\alpha_{0}\left(\frac{\varepsilon }{2}\mu_{0}^{2}\beta_{0}+\frac{\varepsilon }{4}d_{0}^{2}+\frac{\sigma }{2}{∥W^{*}∥}^{2}\right)\;. \end{array}$$

It is clear that *V*, *s* and $\hat{W}\left(t\right)$ are bounded, and hence we obtain $V\geq \frac{s^{2}}{2\beta }$ from Equation (A1), then $s\leq \sqrt{2\beta V}\leq \sqrt{2\beta_{0}V}$. Combining inequality (A5), it yields

(A6) $$\left|s\right|\leq \exp\left(-\frac{t}{2\alpha_{0}}\right)\sqrt{2\beta_{0}V\left(t_{0}\right)}+\sqrt{\alpha_{0}\beta_{0}\left(\varepsilon \mu_{0}^{2}\beta_{0}+\frac{\varepsilon }{2}d_{0}^{2}+\sigma {∥W^{*}∥}^{2}\right)}.$$

This completes the proof. □
